# LONP1 Regulates Mitochondrial Accumulations of HMGB1 and Caspase-3 in CA1 and PV Neurons Following Status Epilepticus

**DOI:** 10.3390/ijms22052275

**Published:** 2021-02-25

**Authors:** Ji-Eun Kim, Hana Park, Tae-Hyun Kim, Tae-Cheon Kang

**Affiliations:** Department of Anatomy and Neurobiology, Institute of Epilepsy Research, College of Medicine, Hallym Unversity, Chuncheon 24252, Korea; jieunkim@hallym.ac.kr (J.-E.K.); M19050@hallym.ac.kr (H.P.); hyun1028@hallym.ac.kr (T.-H.K.)

**Keywords:** apoptosis, caspase-3, epilepsy, HMGB1, LONP1, mitochondrial dynamics, necrosis, seizure

## Abstract

Lon protease 1 (LONP1) is a highly conserved serine peptidase that plays an important role in the protein quality control system in mammalian mitochondria. LONP1 catalyzes the degradation of oxidized, dysfunctional, and misfolded matrix proteins inside mitochondria and regulates mitochondrial gene expression and genome integrity. Therefore, LONP1 is up-regulated and suppresses cell death in response to oxidative stress, heat shock, and nutrient starvation. On the other hand, translocation of high mobility group box 1 (HMGB1) and active caspase-3 into mitochondria is involved in apoptosis of parvalbumin (PV) cells (one of the GABAergic interneurons) and necrosis of CA1 neurons in the rat hippocampus, respectively, following status epilepticus (SE). In the present study, we investigated whether LONP1 may improve neuronal viability to prevent or ameliorate translocation of active caspase-3 and HMGB1 in mitochondria within PV and CA1 neurons. Following SE, LONP1 expression was up-regulated in mitochondria of PV and CA1 neurons. LONP1 knockdown deteriorated SE-induced neuronal death with mitochondrial accumulation of active caspase-3 and HMGB1 in PV cells and CA1 neurons, respectively. LONP1 knockdown did not affect the aberrant mitochondrial machinery induced by SE. Therefore, our findings suggest, for the first time, that LONP1 may contribute to the alleviation of mitochondrial overloads of active caspase-3 and HMGB1, and the maintenance of neuronal viability against SE.

## 1. Introduction

Mitochondria have double membranes and are highly dynamic organelles, whose main function is the production of adenosine triphosphate (ATP) through oxidative phosphorylation. Mitochondria play important roles in cellular homeostasis, such as cell death, Ca^2+^ regulation, and reactive oxygen species (ROS) generation. Since matrix proteins and DNA are located in close proximity to the source of ROS, excessive oxidative ROS production results in denaturation (mutation, abnormal expression, or misfolding) of mitochondrial matrix proteins [1,2]. To counteract oxidized protein denaturation, mitochondrial quality control is very important for maintaining cellular homeostasis to avoid cell damage. Lon protease 1 (LONP1) is a highly conserved serine peptidase that plays an important role in the protein quality control system in mammalian mitochondria. LONP1 belongs to the AAA^+^ (ATPases associated with various cellular activities) family of proteins and requires ATP hydrolysis to degrade proteins [3,4]. LONP1 catalyzes the degradation of oxidized, dysfunctional, and misfolded matrix proteins inside mitochondria and regulates mitochondrial gene expression and genome integrity. Therefore, LONP1 is up-regulated and suppresses cell death in response to oxidative stress, heat shock, and nutrient starvation [5]. Indeed, impairment of LONP1 results in the accumulation of toxic proteins and an extensive apoptotic death via caspase-3 activation, which is associated with a number of neurological diseases [6,7].

Depending on the cellular energetic status, mitochondria structurally change morphologies to exert their functions properly. Two opposing processes are responsible for the prevailing morphologic features of mitochondria: fusion and fission (so-called mitochondrial dynamics). Mitochondrial fusion results in mitochondrial elongation, which is necessary for maintaining and restoring mitochondrial function by facilitating the stochastic redistribution of soluble and membrane components of normal and defective mitochondria. In contrast, fission leads to mitochondrial fragmentation that is useful for the elimination of irreversibly damaged mitochondria. These mitochondrial dynamics not only change their morphologies, but also their mitochondrial and cellular functions [8,9]. For example, aberrant enlarged (giant) mitochondria show swelling, loss of cristae, and destruction of the inner membrane, indicating mitochondrial functional deficiencies such as decreased ATP production [10]. However, excessive mitochondrial fragmentation impairs mitochondrial function and induces releases of cytochrome *c* [11,12].

The activation of the cell death pathway depends on both the triggering stimulus and the cell type [13]. In particular, the hippocampal neurons show the different cell death patterns in response to status epilepticus (SE, a prolonged and uncontrolled seizure activity): parvalbumin (PV) cells (one of the GABAergic interneurons) show apoptotic events with excessive mitochondrial fission, while CA1 neuronal death is necrotic, accompanied by abnormal mitochondrial elongation. Interestingly, these different neuronal death patterns are relevant to the translocation of active caspase-3 (in PV cells) and high mobility group box 1 (HMGB1, in CA1 neurons) into mitochondria, which regulate neuronal apoptosis and necrosis, respectively [14,15,16,17,18]. Caspase-3 is one of the key effectors in the execution of apoptotic cell death. Caspase-3 is synthesized as an inactive proenzyme, which becomes an active enzyme by proteolytic cleavage [19]. The activated caspase-3 rapidly translocates from the cytosol to the mitochondria where it degrades mitochondrial proteins, disintegrates mitochondria, and drives the apoptotic process to completion [12,20]. On the other hand, HMGB1 is a non-histone DNA-binding protein, which stabilizes the nucleosomal structure and facilitates gene transcription [21]. In cells undergoing necrosis, HMGB1 is released from the nucleus to the cytosol [22] and induces a specialized necrosis accompanied by giant mitochondrial formation [23]. Therefore, it is likely that SE-induced aberrant mitochondrial dynamics and/or translocations of active caspase-3 and HMGB1 into mitochondria may affect the patterns of neuronal death in PV cells and CA1 neurons.

Recently, we reported that LONP1 knockdown deteriorates SE-induced neuronal death [24]. Considering the degradation of human mitochondrial transcription factor A (TFAM, a high-mobility group (HMG) protein) by LONP1 [25], it is assumed that the neuroprotective effects of LONP1 may be relevant to the elimination or degradation of the translocated active caspase-3 and HMGB1 in mitochondria, which has been elusive. In the present study, we attempted to explore if LONP1 could improve neuronal viability to prevent or ameliorate translocation of active caspase-3 and HMGB1 in mitochondria within PV cells and CA1 neurons, showing the distinct cell death patterns induced by SE.

Here, we present evidence that LONP1 knockdown deteriorated SE-induced neuronal death with mitochondrial accumulation of active caspase-3 and HMGB1 in PV cells and CA1 neurons, respectively, without affecting the aberrant mitochondrial machinery in these neurons. These findings indicate that LONP1 may eliminate the accumulated active caspase-3 and HMGB1 in mitochondria. Therefore, our findings suggest, for the first time, that LONP1 may contribute to the alleviation of mitochondrial overloads of active caspase-3 and HMGB1, and the maintenance of neuronal viability against SE.

## 2. Results

### 2.1. SE-Induced Mitochondrial Caspase-3 and HMGB1 Translocations in PV and CA1 Neurons, Respectively

**Figure 1:**
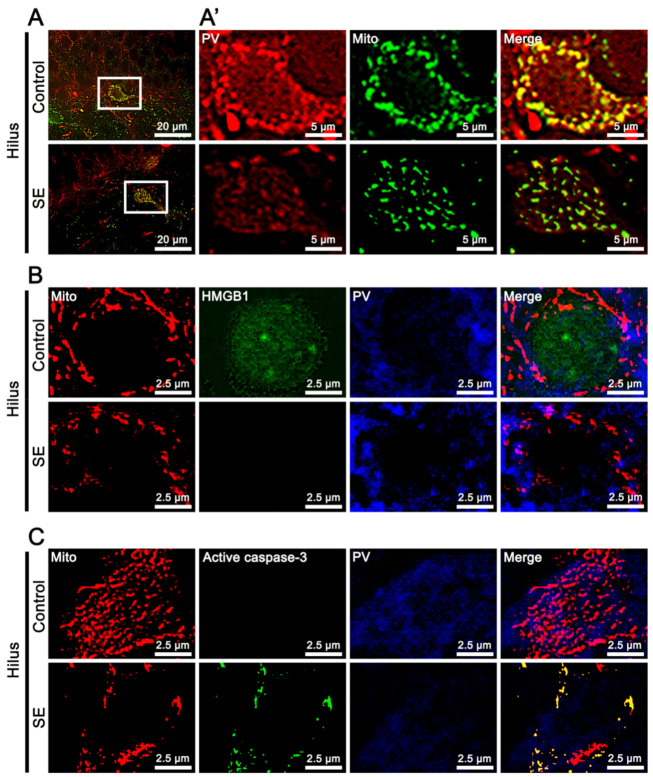
Mitochondrial accumulations of high mobility group box 1 (HMGB1) and active caspase-3 in parvalbumin (PV) neurons following status epilepticus (SE). (**A**) Reductions in PV expression and mitochondrial length in the hilus. (**A’**) shows high magnifications of rectangles in the left panels. (**B**) Absence of translocation of HMGB1 from nucleus to mitochondria (Mito) accumulation in PV neurons following SE. (**C**) Mitochondrial active caspase-3 in PV neurons following SE.

**Figure 2:**
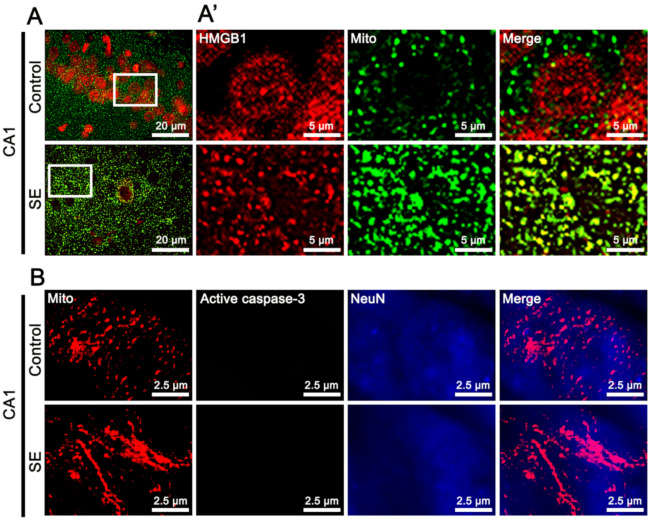
Mitochondrial accumulations of HMGB1 and active caspase-3 in neuron nuclei antigen (NeuN)-positive CA1 neurons following SE. (**A**) Translocation of HMGB1 from nucleus to mitochondria in CA1 neurons following SE. (**A’**) shows high magnifications of rectangles in the left panels. (**B**) Absence of mitochondrial accumulation of active caspase-3 in CA1 neurons following SE.

Figure 1 shows that SE reduced PV expression and decreased the mitochondrial length in PV cells, as compared to controls (Figure 1A). In addition, SE resulted in HMGB1 release from the nucleus in PV cells, which was rarely detected in fragmented mitochondria (Figure 1B). In contrast, active caspase-3 levels were elevated in fragmented mitochondria following SE (Figure 1C). In the CA1 neurons, SE elongated mitochondrial length and accumulated HMGB1 in mitochondria (Figure 2A). However, SE did not induce active caspase-3 translocation into mitochondria (Figure 2B). These findings indicate that SE may induce mitochondrial accumulations of HMGB1 and active caspase-3 in neuronal-specific manners.

### 2.2. Effects of LONP1 Knockdown on SE-Induced Neuronal Death

**Figure 3:**
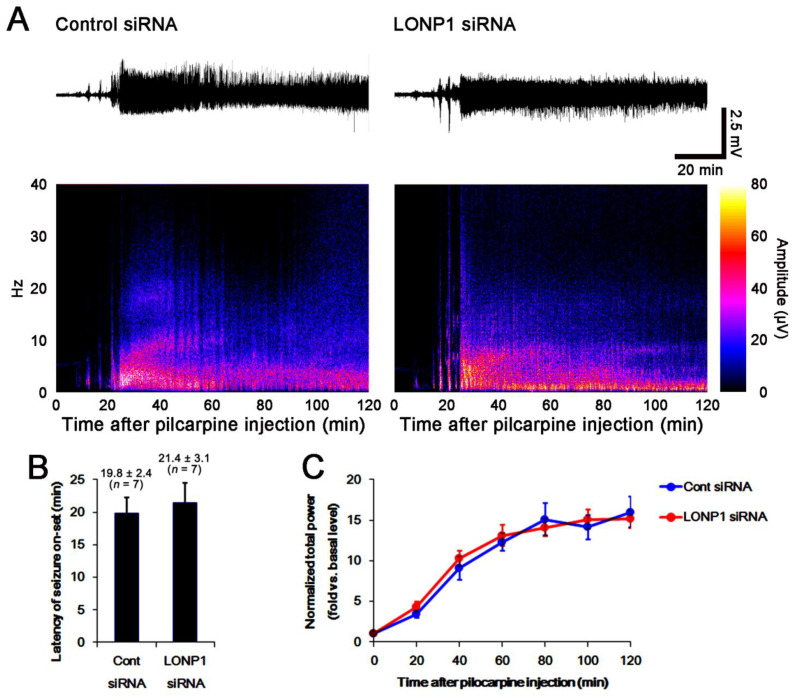
Effects of control siRNA and LONP1 siRNA on seizure activity in response to pilocarpine. As compared to control siRNA (Cont siRNA), LONP1 siRNA does not affect seizure activity induced by pilocarpine. (**A**) Representative EEG traces and frequency power spectral temporal maps in response to pilocarpine. (**B**,**C**) Quantification of the latency of seizure onset ((**B**), mean ± S.E.M.; Student’s *t*-test) and total EEG power (seizure intensity) in response to pilocarpine ((**C**), mean ± S.E.M.; *n* = 7; repeated measures ANOVA).

**Figure 4:**
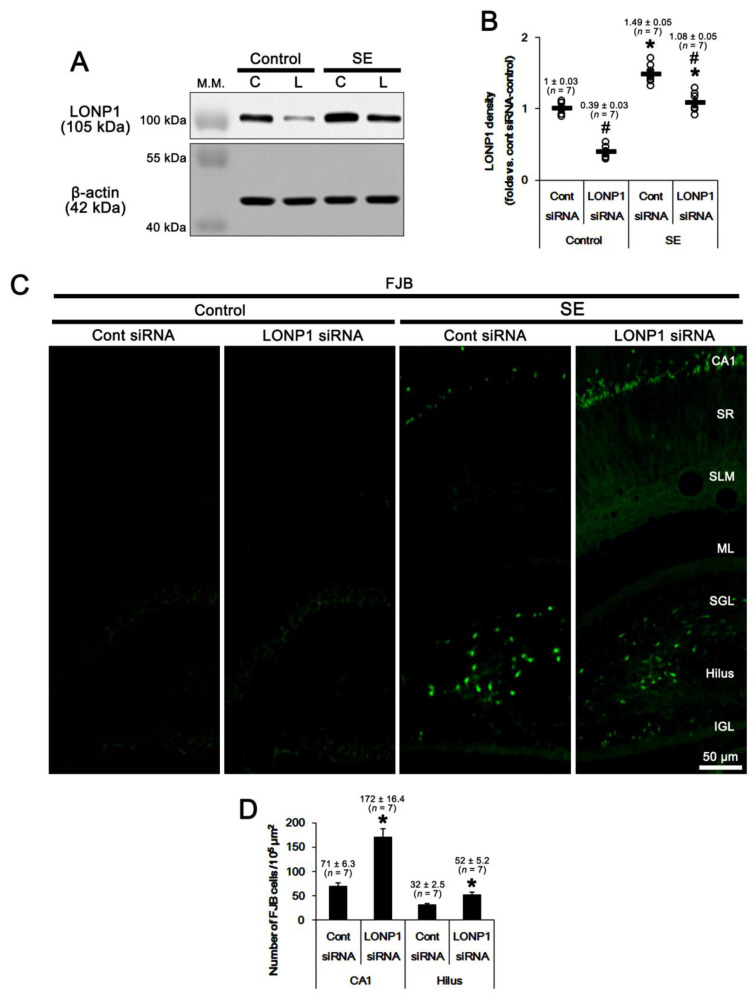
Effects of control siRNA and LONP1 siRNA on LONP1 expression and neuronal death in response to pilocarpine. SE increases LONP1 expression level. As compared to control siRNA (Cont siRNA, C), LONP1 siRNA (L) decreases LONP1 protein level under physiological and post-SE condition. (**A**) Representative Western blots of LONP1 protein level in the whole hippocampus (M.W. marker, molecular weight marker). (**B**) Quantification of LONP1 protein level based on Western blot data. Open circles indicate each individual value. Horizontal bars indicate mean value (mean ± S.E.M.; *^,#^ *p* < 0.05 vs. control animals and control siRNA, respectively; one-way ANOVA). (**C**) Representative Fluoro-Jade B (FJB) staining images. LONP1 siRNA exacerbates SE-induced degenerations of CA1 neurons and hilus (including PV) interneurons, presumably including PV cells. Abbreviations: CA1, CA1 pyramidal cell layer; SR, stratum radiatum; SLM, stratum lacunosum-moleculare; ML, the molecular layer of the dentate gyrus; SGL, superior dentate granule cell layer; IGL, inferior dentate granule cell layer. (**D**) Quantification of the number of FJB-positive CA1- and hilus interneurons, presumably including PV cells, following SE (mean ± S.E.M.; * p < 0.05 vs. control siRNA; Student’s *t*-test). Abbreviations: CA1, CA1 neurons; Hilus, hilus interneurons, presumably including PV cells.

To evaluate the role of LONP1 in SE-induced neuronal death, we applied LONP1 siRNA prior to SE induction. As compared to control siRNA, LONP1 knockdown did not affect the latency of seizure onset and the total electroencephalogram (EEG) power in response to pilocarpine (Figure 3A–C). In control animals, LONP1 siRNA reduced the LONP1 protein level to 0.4-fold of control siRNA in the whole hippocampus (*F*_(1,12)_ = 184, *p* < 0.001 vs. control siRNA, one-way ANOVA; Figure 4A,B and Figure S1). SE increased LONP1 expression to 1.49-fold of control animal levels (*F*_(1,12)_ = 193.5, *p* < 0.001 vs. control animals, one-way ANOVA; Figure 4A,B), which was attenuated by LONP1 siRNA (*F*_(1,12)_ = 29.9, *p* < 0.001 vs. control siRNA, one-way ANOVA; Figure 4A,B and Figure S1). In addition, Fluoro-Jade B (FJB) staining (indicative of degenerating neurons) showed that LONP1 knockdown aggravated SE-induced degeneration of CA1-(*F*_(1,12)_ = 32.8, *p* < 0.001 vs. control siRNA, one-way ANOVA) and hilus interneurons, presumably including PV cells (*F*_(1,12)_ = 12.9, *p* = 0.004 vs. control siRNA, one-way ANOVA) (Figure 4C,D). These findings indicate that up-regulation of LONP1 may play a protective role against SE insults.

### 2.3. Effects of LONP1 Knockdown on Mitochondrial LONP1 Level and Mitochondrial Dynamics Following SE

**Figure 5:**
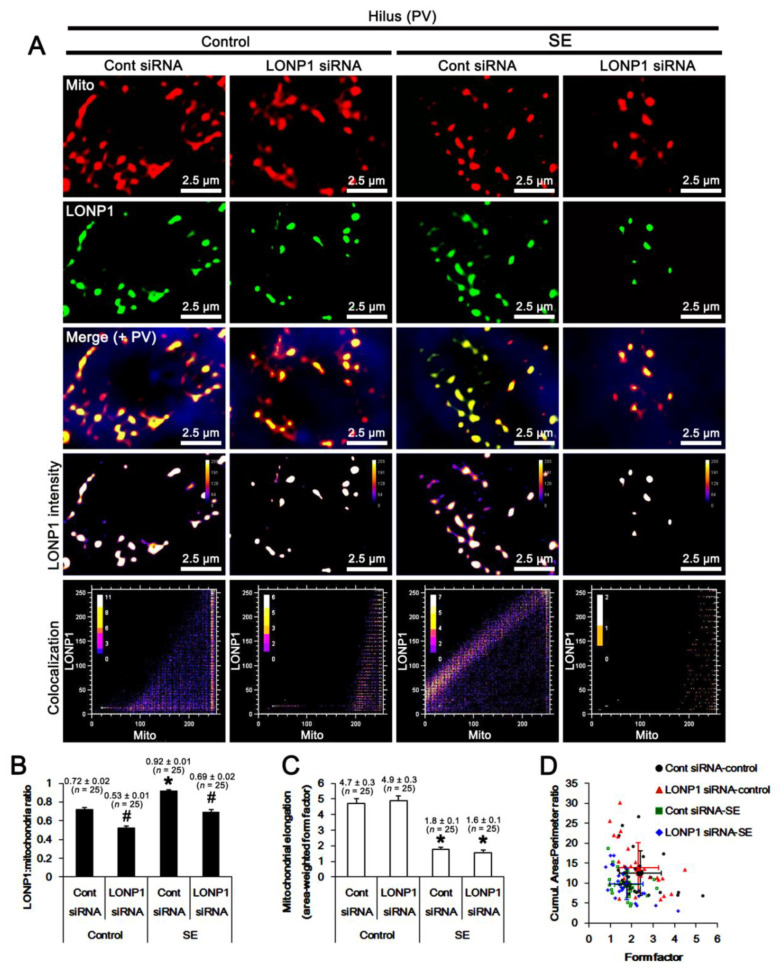
Effects of control siRNA and LONP1 siRNA on LONP1 expression and mitochondrial dynamics in PV neurons following SE. As compared to control siRNA (Cont siRNA), LONP1 siRNA decreases LONP1 protein level under physiological and post-SE conditions. (**A**) Representative photos of LONP1 expression, intensity, and the degree of colocalization in mitochondria within PV cells. (**B**) Quantification of the LONP1/mitochondria ratio (the degree of colocalization) following SE (mean ± S.E.M.; *^,#^ *p* < 0.05 vs. control animals and control siRNA, respectively; one-way ANOVA). (**C**) Quantification of mitochondrial elongation index (area-weighted form factor) following SE (mean ± S.E.M.; * p < 0.05 vs. control animals; one-way ANOVA). (**D**) Quantification of the cumulative area/perimeter ratio (indicative of the transition from elongated, isolated mitochondria to a reticular network or aggregation of interconnected mitochondria; *F*_(1,48)_ = 4.6, *p* = 0.037 vs. control animals, one-way ANOVA) and the form factor (a parameter of the transition from the sphere to elongated, complex-shaped, but still isolated mitochondria; *F*_(1,48)_ = 6.5, *p* = 0.014 vs. control animals, one-way ANOVA) following SE. LONP1 knockdown does not affect the cumulative area/perimeter ratio and the form factor in PV cells under physiological and post-SE conditions. Each small and open symbol represents average shape metrics from one neuron (*n* = total 25 cells in each group). Large and filled symbols are population averages from each group (mean ± S.D.; *n* = 5).

Next, we evaluated the effect of LONP1 knockdown on mitochondrial dynamics. In PV cells, the LONP1/mitochondria ratio was 0.72 under physiological conditions, which was decreased to 0.53 by LONP1 siRNA (*F*_(1,48)_ = 62.5, *p* < 0.001 vs. control siRNA, one-way ANOVA; Figure 5A,B). Following SE, LONP1/mitochondria was elevated to 0.92 (*F*_(1,48)_ = 78.5, *p* < 0.001 vs. control animals, one-way ANOVA; Figure 5A,B), which was decreased to 0.69 by LONP1 knockdown (*F*_(1,48)_ = 70.7, *p* < 0.001 vs. control siRNA, one-way ANOVA; Figure 5A,B). SE led to a 0.37-fold decrease in mitochondrial elongation (represented as area-weighted form factor [26,27]) in PV cells (*F*_(1,48)_ = 73.2, *p* < 0.001 vs. control animals, one-way ANOVA). Mitochondrial elongation was unaffected by LONP1 siRNA under physiological and post-SE conditions (Figure 5A,C). SE reduced the cumulative area/perimeter ratio (indicative of the transition from elongated, isolated mitochondria to a reticular network or aggregation of interconnected mitochondria [26,27]) to 0.77-fold of control animal levels (*F*_(1,48)_ = 4.6, *p* = 0.037 vs. control animals, one-way ANOVA) and the form factor (a parameter of the transition from the sphere to elongated, complex-shaped, but still isolated mitochondria [26,27]) to 0.75-fold of control animal levels (*F*_(1,48)_ = 6.5, *p* = 0.014 vs. control animals, one-way ANOVA; Figure 5A,D). Neither the cumulative area/perimeter ratio nor the form factor was influenced by LONP1 knockdown (Figure 5A,D). These findings indicate that SE may result in excessive mitochondrial fission in PV cells, independently of the altered LONP1 level.

**Figure 6:**
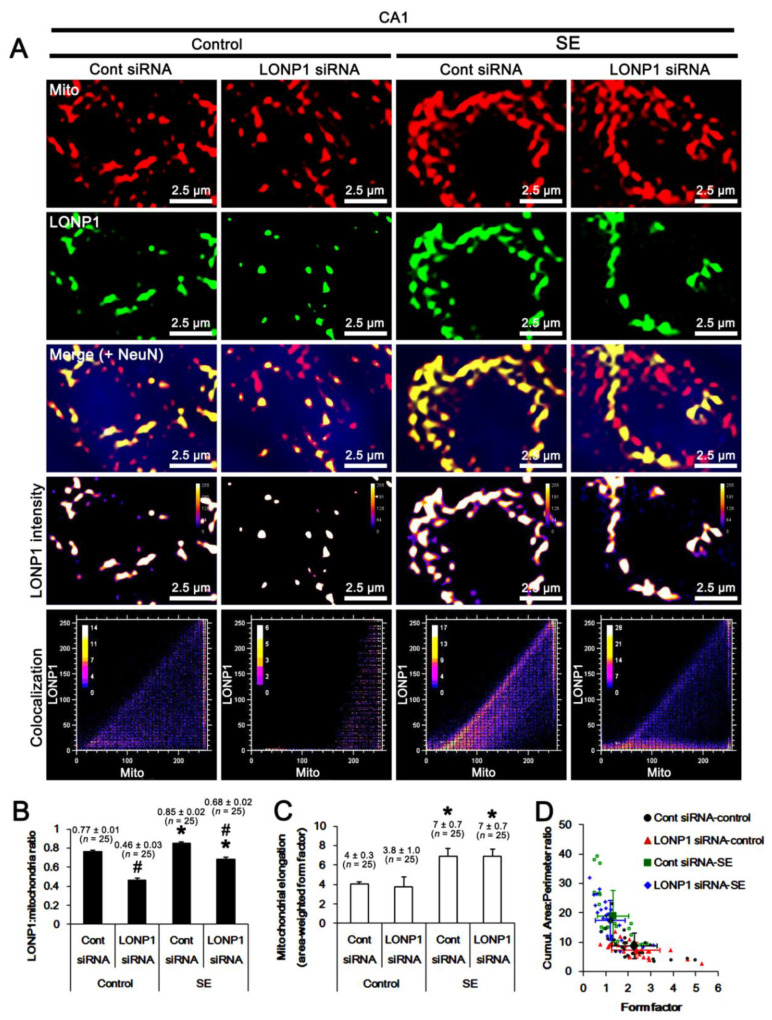
Effects of control siRNA and LONP1 siRNA on LONP1 expression and mitochondrial dynamics in CA1 neurons following SE. As compared to control siRNA (Cont siRNA), LONP1 siRNA decreases LONP1 protein level under physiological and post-SE conditions. (**A**) Representative photos of LONP1 expression, intensity, and the degree of colocalization in mitochondria within PV cells. (**B**) Quantification of the LONP1/mitochondria ratio following SE (mean ± S.E.M.; *^,#^ *p* < 0.05 vs. control animals and control siRNA, respectively; one-way ANOVA). (**C**) Quantification of mitochondrial elongation index following SE (mean ± S.E.M.; * p < 0.05 vs. control animals; one-way ANOVA). (**D**) Quantification of the cumulative area/perimeter ratio (*F*_(1,48)_ = 25.7, *p* < 0.001 vs. control animals, one-way ANOVA) and the form factor (*F*_(1,48)_ = 14.2, *p* < 0.001 vs. control animals, one-way ANOVA) following SE. LONP1 knockdown does not affect the cumulative area/perimeter ratio and the form factor in CA1 neurons under physiological and post-SE conditions. Each small and open symbol represents average shape metrics from one neuron (*n* = total 25 cells in each group). Large and filled symbols are population averages from each group (mean ± S.D.; *n* = 5).

In CA1 neurons, the LONP1/mitochondria ratio was 0.77 under physiological conditions, which was reduced to 0.46 by LONP1 siRNA (*F*_(1,48)_ = 89.8, *p* < 0.001 vs. control siRNA, one-way ANOVA; Figure 6A,B). Unlike PV cells, the LONP1/mitochondria ratio was increased to 0.85 following SE (*F*_(1,48)_ = 19.8, *p* < 0.001 vs. control animals, one-way ANOVA; Figure 6A,B), which was decreased to 0.68 by LONP1 knockdown (*F*_(1,48)_ = 44.4, *p* < 0.001 vs. control siRNA, one-way ANOVA; Figure 6A,B). SE resulted in a 1.75-fold increase in mitochondrial elongation (*F*_(1,48)_ = 13.5, *p* < 0.001 vs. control animals, one-way ANOVA; Figure 6A,C), which was unaffected by LONP1 siRNA under physiological and post-SE conditions (Figure 6A,C). SE also increased the cumulative area/perimeter ratio to 2-fold of control animal levels (*F*_(1,48)_ = 25.7, *p* < 0.001 vs. control animals, one-way ANOVA), while it reduced the form factor to 0.6-fold of control animal levels (*F*_(1,48)_ = 14.2, *p* < 0.001 vs. control animals, one-way ANOVA; Figure 6A,D). Both the cumulative area/perimeter ratio and the form factor were unaffected by LONP1 knockdown (Figure 6A,D). These findings indicate that SE may lead to mitochondrial elongation and their aggregation, indicating aberrant mitochondrial fusion, in CA1 neurons, and that LONP1 may not be involved in these phenomena. Taken together, our findings suggest that the up-regulated LONP1 expression may not participate in aberrant mitochondrial dynamics in PV and CA1 neurons following SE.

### 2.4. Effects of LONP1 Knockdown in Mitochondrial Accumulation of Active Caspase-3 in PV Cells Following SE 

**Figure 7:**
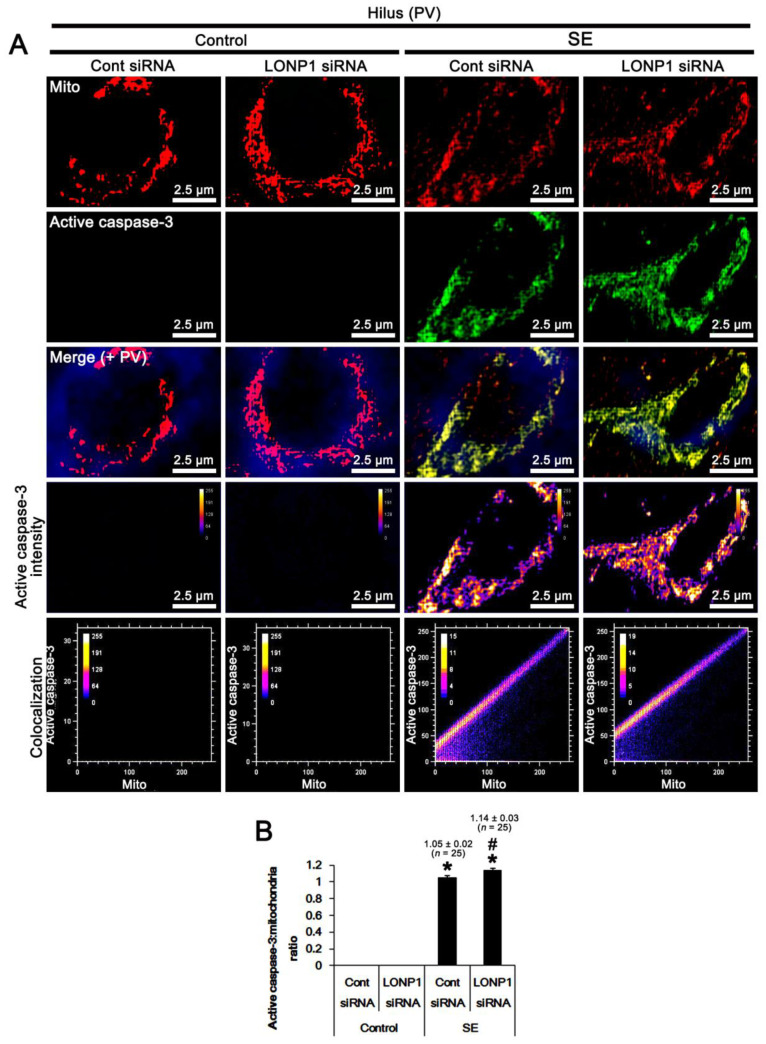
Effects of control siRNA and LONP1 siRNA on mitochondrial accumulation of active caspase-3 in PV neurons following SE. As compared to control siRNA (Cont siRNA), LONP1 siRNA increases mitochondrial accumulation of active caspase-3 following SE. (**A**) Representative photos of active caspase-3 level, intensity, and the degree of colocalization in mitochondria within PV cells. (**B**) Quantification of the active caspase-3/mitochondria ratio (the degree of colocalization) following SE (mean ± S.E.M.; *^,#^ *p* < 0.05 vs. control animals and control siRNA, respectively; one-way ANOVA).

Since SE led to mitochondrial accumulation of active caspase-3 (Figure 1), we investigated the effect of LONP1 knockdown on it. Under physiological conditions, the mitochondrial active caspase-3 signal was rarely detected in PV cells, which was unaffected by LONP1 knockdown (Figure 7A,B). However, SE obviously increased the active capase-3/mitochondria ratio to 1.05 (*F*_(1,48)_ = 2202.6, *p* < 0.001 vs. control animals, one-way ANOVA; Figure 7A,B). LONP1 knockdown increased the active capase-3/mitochondria ratio to 1.14 (*F*_(1,48)_ = 5.7, *p* = 0.021 vs. control siRNA, one-way ANOVA; Figure 7A,B). These findings indicate that the up-regulation of mitochondrial LONP1 may be involved in the eliminations of accumulated active caspase-3 in mitochondria following SE.

### 2.5. Effects of LONP1 Knockdown on Mitochondrial HMGB1 Accumulation in CA1 Neurons Following SE

**Figure 8:**
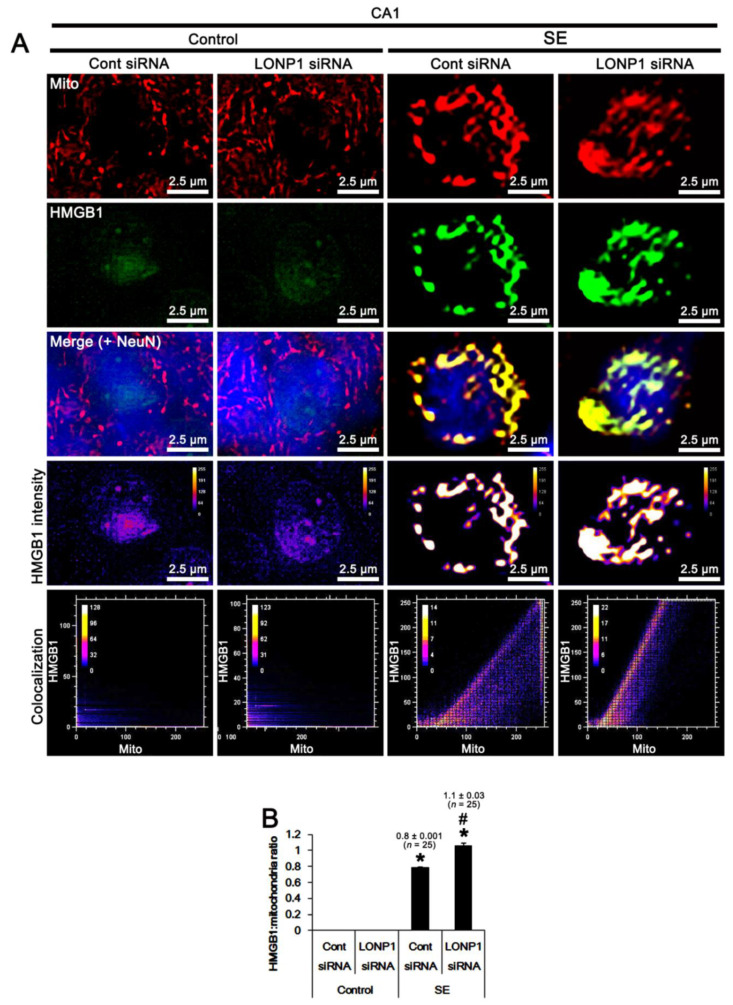
Effects of control siRNA and LONP1 siRNA on mitochondrial HMGB1 accumulation in CA1 neurons following SE. As compared to control siRNA (Cont siRNA), LONP1 siRNA increases mitochondrial HMGB1 translocation following SE. (**A**) Representative photos of level, intensity, and the degree of colocalization in mitochondria within CA1 neurons. (**B**) Quantification of the HMGB1/mitochondria ratio (the degree of colocalization) following SE (mean ± S.E.M.; *^,#^ *p* < 0.05 vs. control animals and control siRNA, respectively; one-way ANOVA).

Since, in the present study, we also found that SE led to HMGB1 translocation from the nucleus to mitochondria (Figure 2), we validated the effect of LONP1 knockdown on mitochondrial HMGB1 accumulation induced by SE. Under physiological conditions, mitochondrial HMGB1 accumulation was rarely detected in CA1 neurons, which was unaffected by LONP1 knockdown (Figure 8A,B). However, SE apparently increased the HMGB1/mitochondria ratio to 0.8 (*F*_(1,48)_ = 6620, *p* < 0.001 vs. control animals, one-way ANOVA; Figure 8A,B). LONP1 knockdown further elevated the HMGB1/mitochondria ratio to 1.1 (*F*_(1,48)_ = 97.5, *p* < 0.001 vs. control animals, one-way ANOVA; Figure 8A,B). These findings indicate that HMGB1 may be one of the substrates of LONP1, and that the mitochondrial LONP1 up-regulation may be an adaptive response for clearance of mitochondrial HMGB1 accumulation following SE.

## 3. Discussion

LONP1 emerges as a major regulator of multiple mitochondrial functions. LONP1 catalyzes the degradation of oxidatively modified matrix proteins, chaperones the assembly of inner membrane complexes, and participates in the regulation of mitochondrial gene expression and genome integrity. Impairment of LONP1 results in the accumulation of toxic oxidized proteins in the brain, which can cause severe neuronal damage [7]. Consistent with our previous study [24,28], the present data show that SE up-regulated the mitochondrial LONP1 level in PV and CA1 neurons, while LONP1 knockdown did not affect the viability and mitochondrial dynamics in both neurons under physiological and post-SE conditions. Since LONP1 plays a direct role in the turnover of isoform 1 of COX subunit 4 (COX4-1) to COX4-2 for enhancing mitochondrial respiration [29], LONP1 may act as one of the important housekeeping enzymes by maintaining mitochondrial respiration at tolerable levels under pathological conditions, independent of mitochondrial dynamics. Indeed, dysfunction of LONP1 in mitochondria has been associated with a number of neurological disorders including Parkinson’s disease, Friedreich ataxia, and amyotrophic lateral sclerosis [24,28,30,31,32]. In particular, LONP1 downregulation results in extensive (although not universal) apoptosis with the classic hallmark of caspase-3 activation. Therefore, LONP1 functions seem to be mostly involved in apoptosis. This is because the defects of proteolytic LONP1 activity would cause accumulation of aggregated proteins inside mitochondria, and loss of its chaperone function impairs mitochondrial respiration and membrane potential, rendering cells susceptible to apoptotic stimuli [6]. In the present study, LONP1 knockdown did not lead to apoptosis in PV and CA1 neurons under physiological conditions. However, it increased the mitochondrial active caspase-3 level in PV cells following SE. These findings indicate that following SE, the accumulation of abnormal matrix proteins induced by LONP1 dysfunctions would facilitate active caspase-3 import in PV cells, or that loss of LONP1 could not catalyze excessive active caspase-3 in these cells. Both cases indicate that the impaired LONP1 protease activity may lead to mitochondrial active caspase-3 overloads in PV cells. The imported active caspase-3 activates residual or other resident procaspases in the organelle, which would establish a positive feedback amplification mechanism. In addition, active caspase-3 degrades mitochondrial proteins such as the mitochondrial respiratory chain protein complexes, disintegrates mitochondrial integrity and mitochondrial DNA (mtDNA), and triggers cytochrome *c* release and generation of reactive oxygen species [12,20]. However, the underlying mechanisms are unknown, and thus our findings provide new clues that at least LONP1 may regulate the accumulation of active caspase-3 in mitochondria under pathological conditions, which would decrease the rate of apoptotic cell death.

In the present study, we found that LONP1 knockdown increased mitochondrial HMGB1 accumulation in CA1 neurons following SE. Since LONP1 degrades TFAM (an HMG protein) [25], it is likely that LONP1 may also degrade imported HMGB1 into mitochondria. In various cells, nucleocytoplasmic HMGB1 release is observed during necrosis [14,22,33]. Traditionally, HMGB1 is released from the nucleus through chromosome region maintenance 1 (CRM1) during necrosis and subsequently secreted into the extracellular space, which evokes potentially inflammatory responses via toll-like receptor 4 (TLR4) and/or receptor for advanced glycan endproducts (RAGE) [22,34,35]. Furthermore, mitochondria are permeable to HMGB1 released from the nucleus [16,36]. Although HMGB1 function in mitochondria remains poorly understood, several possible hypotheses are considerable. Since HMGB1 rescues the impairment of mitochondrial function and involves mitochondrial reorganization [37,38], it is likely that the mitochondrial HMGB1 imports would be one of the compensatory responses for the maintenance of mitochondrial functions. However, translocation of HMGB1 to mitochondria facilitates and deteriorates necrotic CA1 neuronal death induced by SE without altering the mitochondrial machinery. Furthermore, leptomycin B (LMB, a CRM1 inhibitor) attenuates SE-induced CA1 neuronal death accompanied by abolishing the mitochondrial HMGB1 imports [16]. HMGB1 contributes to mitochondrial oxidative damage [39]. HMGB1 also interacts with mtDNA and acts as damage associated molecular patterns (DAMPs), which activates the TLR9 signaling pathway [40]. The HMGB1/RAGE axis also inhibits mitophagy flux, which plays an important role in the mitochondrial quality control by removing abnormal/aberrant mitochondria [41]. Considering these HMGB1-mediated mitochondrial defects, our findings suggest that LONP1 may play a protective role against SE-induced CA1 neuronal death.

What makes the selective HMGB1 and active caspase-3 translocations into elongated mitochondria (in CA1 neurons), but not fragmented mitochondria (in PV cells)? Since active caspase-3 is rarely observed in CA1 neurons, the inability to induce caspase-3 would be a reason for the absence of mitochondrial accumulation of active caspase-3 in these neurons. Furthermore, WY14643 (an activator of mitochondrial fission) induces mitochondrial accumulation of active caspase-3 in CA1 neurons under physiological conditions, while it cannot evoke neuronal death [18]. Thus, it is likely that mitochondrial fission may be required for caspase-3 activation. However, nuclear HMGB1 signals were obviously detected in both PV cells and CA1 neurons. Although the underlying mechanisms are unveiled in the present study, a possible factor could be speculated. Since the molecular weight of HMGB1 is ~30 kDa [42], while that of active caspase-3 is 12~17 kDa [43], the larger surface areas (by mitochondrial elongation) would be required for the penetration of higher molecular weights of HMGB1 across mitochondrial membranes, if active transport system is absent. Indeed, mitochondrial HMGB1 translocation is enhanced by Mdivi-1 (an inhibitor of mitochondrial fission), but abrogated by WY14643 [18]. Furthermore, exogenous HMGB1 enters the mitochondria, which is followed by the formation of giant mitochondria independently of HMGB1 receptors such as TLR4 or RAGE [23]. Thus, our findings suggest that the mitochondrial elongations may facilitate HMGB1 imports into mitochondria.

On the other hand, increased LONP1 interacts with p53 in the mitochondrial matrix and restrains the apoptosis induced by p53 under oxidative stress by rescuing the loss of the mitochondrial membrane potential [44]. Furthermore, collapse of the mitochondrial membrane potential also results in necrosis by excessive ROS production [45]. Although we could not directly examine it in the present study, it is likely that LONP1 knockdown may lead to the impairment of the mitochondrial membrane potential, which would also accelerate neuronal death induced by ROS. Further studies are needed to elucidate the effect of LONP1 knockdown on the mitochondrial membrane potential following SE induction. 

## 4. Materials and Methods 

### 4.1. Experimental Animals and Chemicals

Male Sprague-Dawley (SD) rats (7 weeks old, *n* = 61) were kept under controlled environmental conditions (23–25 °C, 12 h light/dark cycle). Rats freely accessed water and standard laboratory food during the experiment. All animal procedures were approved and carried out according to the Administrative Panel on Laboratory Animal Care of Hallym University (the authorization number: Hallym 2018-2, 26 April 2018). All possible efforts were taken to avoid animals’ suffering and to minimize the number of animals used during the experiment. All reagents were obtained from Sigma-Aldrich (St. Louis, MO, USA), except as noted.

### 4.2. LONP1 Knockdown and Electrode Implantation

Rats were anesthetized with Isoflurane anesthesia (3% induction, 1.5–2% for surgery, and 1.5% maintenance, in a 65:35 mixture of N_2_O:O_2_) and placed in a stereotaxic apparatus. Thereafter, animals were implanted with a brain infusion kit 1 (Alzet, Cupertino, CA, USA) into the right lateral ventricle. The following coordinates were used (from bregma): 1 mm posterior; 1.5 mm lateral; 3.5 mm depth. The infusion kit was sealed with dental cement and connected to an osmotic pump (1007D, Alzet, Cupertino, CA, USA). Each osmotic pump contained: (1) non-targeting control siRNA (5-GCAACUAACUUCGUUAGAAUCGUUAUU-3) or (4) LONP1 siRNA (5-GAGACAAGUUGCGCAUGAUTT-3). An osmotic pump was placed in a subcutaneous pocket between scapulas. To evaluate the effect of LONP1 knockdown on seizure susceptibility, some animals were also implanted with a stainless-steel electrode (Plastics One, Roanoke, VA, USA) into the left dorsal hippocampus (−3.8 mm posterior; 2.0 mm lateral; −2.6 mm depth). The connecting wire and electrode socket were then inserted in an electrode pedestal (Plastics One, Roanoke, VA, USA) and secured to the exposed skull with dental acrylic.

### 4.3. SE Induction and EEG Analysis

Two days after surgery, rats were given 127 mg/kg LiCl. Twenty-four hours after LiCl treatment, SE was induced by a single dose (30 mg/kg) of pilocarpine. To block the peripheral effect of pilocarpine, atropine methylbromide (5 mg/kg i.p.) was injected into animals 20 min prior to pilocarpine injection. As controls, rats were treated with saline instead of pilocarpine. In electrode-implanted animals, EEG signals were recorded with a DAM 80 differential amplifier (0.1–3000 Hz bandpass, World Precision Instruments, Sarasota, TL, USA), digitized (sampling rates, 1000 Hz), and analyzed using LabChart Pro v7 (AD Instruments, Bella Vista, NSW, Australia). Total EEG power and spectrograms were automatically calculated in 2-hour recording session using a Hanning sliding window with 50% overlap. Two hours after SE, animals received diazepam (Valium; Roche, France; 10 mg/kg, i.p.) to terminate SE.

### 4.4. Tissue Processing

Three days after SE, animals were transcardially perfused with 4% paraformaldehyde under urethane anesthesia (1.5 g/kg i.p.) and after additional fixation overnight at 4 °C. The brains were rinsed in PB containing 30% sucrose at 4 °C for 2 days. Thereafter, coronal sections (30 μm) were cut with a cryostat. For Western blot, animals were decapitated under urethane anesthesia (1.5 g/kg, i.p.). The hippocampus was rapidly selected and homogenized in lysis buffer. The protein concentration in the supernatant was calibrated using a Micro BCA Protein Assay Kit (Pierce Chemical, Rockford, IL, USA).

### 4.5. Western Blot

Western blotting was performed according to standard procedures. Briefly, tissue lysate proteins were blotted onto membranes (Schleicher and Schuell BioScience Inc., Keene, NH, USA) and then incubated with the primary antibodies shown in Table 1. Immunoreactive bands were detected and quantified on an ImageQuant LAS4000 system (GE Healthcare Korea, Seoul, South Korea). The values of each sample were normalized with the amount of β-actin level.

### 4.6. Immunohistochemistry and FJB Staining

After blockade with 10% goat serum (Vector, Burlingame, CA, USA) in PBS for 30 min at room temperature, free-floating coronal sections (fifteen tissue sections/well) were incubated with the mixture of primary antibodies (Table 1) in PBS containing 0.3% triton X-100 at room temperature overnight. After three washes in PBS, fluorescein isothiocyanate (FITC)-, Cy3-, or aminomethylcoumarin acetate (AMCA)-conjugated secondary antibodies (Vector, Burlingame, CA, USA) were applied for 1 h at room temperature. Brain sections incubated with preimmune serum or second antibody alone were used as negative controls. To analyze the neuronal damage, we applied Fluoro-Jade B (FJB) staining (Histo-Chem Inc., Jefferson, AR, USA), according to the manufacturer’s instructions.

### 4.7. Cell Count and Mitochondrial Morphometry

The hippocampal tissues were captured (10 sections per animal), and areas of interest (1 × 10^5^ μm^2^) were selected from the CA1 region and hilus. Thereafter, FJB-positive neurons were counted on 20× images using AxioVision Rel. 4.8 Software. In addition, five brain sections from each animal (five animals each in the control siRNA-treated control group, control siRNA-treated SE-experienced group, LONP1 siRNA-treated control group, and LONP1 siRNA-treated SE-experienced group) were randomly selected at different rostro-caudal hippocampal levels. One randomly selected CA1 or PV neuron from each slice (total 25 cells in each group) was used for quantification of mitochondrial morphometry and each fluorescent intensity using AxioVision Rel. 4.8 and ImageJ software. Mitochondria were analyzed for perimeter and area. Mitochondrial parameters were calculated as follows: area-weighted form factor = perimeter^2^/4π (indicative of mitochondrial elongation); form factor = perimeter^2^/4π × area (indicating the transition from punctiform to elongated, complex-shaped, but still isolated mitochondria); cumulative area/perimeter ratio = Σarea/Σperimeter (indicating the transition from elongated, isolated mitochondria to a reticular network or aggregation of interconnected mitochondria) [26,27]. The investigators were blinded to experimental groups in performing morphological analysis and immunohistochemical experiments.

### 4.8. Statistical Analysis

Data were analyzed by two-tailed Student’s *t*-test, one-way repeated measure ANOVA, or one-way ANOVA followed by Bonferroni post hoc tests for multiple comparisons. All analyses were performed blind to the experimental conditions.

## 5. Conclusions

In the present study, we demonstrated that active caspase-3 and HMGB1 were preferentially accumulated into fragmented (in PV cells) and elongated mitochondria (in CA1 neurons) following SE, respectively. In addition, LONP1 knockdown accelerated the mitochondrial accumulations of active caspase-3 and HMGB1 in each neuronal population. These overloads were relevant to the SE-induced neuronal death without altering aberrant mitochondrial dynamics in both neurons. Therefore, our findings suggest that LONP1 may ameliorate the accumulations of active caspase-3 and HMGB1 in mitochondria and be practically involved in the abnormal mitochondrial machinery-mediated neuronal death induced by SE.

## Figures and Tables

**Table 1:** Primary antibodies used in the present study.

Antigen	Host	Manufacturer (Catalog Number)	Dilution Used
Active caspase-3	Rabbit	Cell signaling (#9664)	1:400 (IH)
High mobility group box 1 (HMGB1)	Rabbit	Abcam (#ab18256)	1:100 (IH)
Lon protease 1 (LONP1)	Rabbit	Proteintech (15440-1-AP)	1:100 (IH) 1:1000 (WB)
Mitochondrial marker (Mitochondrial complex IV subunit 1, MTCO1)	Mouse	Abcam (#ab14705)	1:500 (IH)
NeuN	Guinea pig	Millipore (#3238431)	1:1000 (IH)
Parvalbumin (PV)	Goat	Swant (#PVG213)	1:100,000 (IH)
β-actin	Mouse	Sigma (A5316)	1:5000 (WB)

IH, immunohistochemistry; WB, Western blot.

## Data Availability

Not applicable.

## Supplementary Materials

Supplementary Materials can be found at https://www.mdpi.com/1422-0067/22/5/2275/s1.

## Abbreviations

HMGB1	High mobility group box 1
LONP1	Lon protease 1
PV	Parvalbumin
SE	Status epilepticus
