# Supplementary material for: LONP1 Regulates Mitochondrial Accumulations of HMGB1 and Caspase-3 in CA1 and PV Neurons Following Status Epilepticus
Source: Int J Mol Sci. 2021 Feb 25;22(5):2275. doi: 10.3390/ijms22052275 (PMC7956547; doi:10.3390/ijms22052275)
Supplement: Supplementary file 1 [file ijms-22-02275-s001.pdf]

Supplementary information

LONP1 regulates mitochondrial accumulations of HMGB1 and caspase-3 in CA1 and PV neurons following status epilepticus

Ji-Eun Kim,[1] Hana Park,[1] Tae-Hyun Kim,[1] Tae-Cheon Kang[1,*]

[1] Department of Anatomy and Neurobiology, Institute of Epilepsy Research, College of Medicine, Hallym University, Chuncheon 24252, South Korea; jieunkim@hallym.ac.kr (J.-E.K.); M19050@hallym.ac.kr (H.P.); hyun1028@hallym.ac.kr (T.-H.K.).

* Correspondence: tckang@hallym.ac.kr; Tel.:+82-33-248-2524; Fax: +82-33-248-2525.

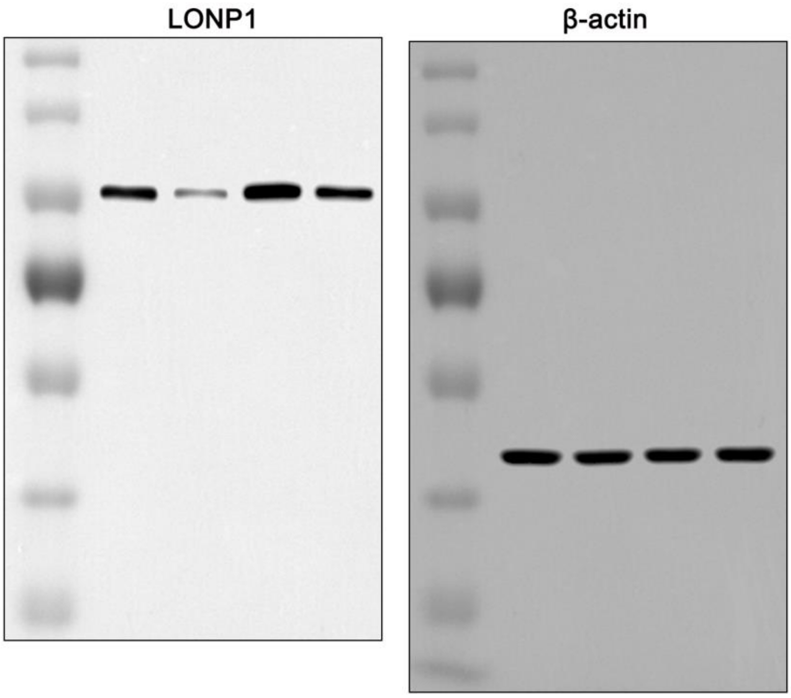

Supplementary Figure 1. Representative full-gel images of Western blots in Figure 4A.
